# Au_2_phen and Auoxo6, Two Dinuclear Oxo-Bridged Gold(III) Compounds, Induce Apoptotic Signaling in Human Ovarian A2780 Cancer Cells

**DOI:** 10.3390/biomedicines9080871

**Published:** 2021-07-23

**Authors:** Giulia Gorini, Francesca Magherini, Tania Fiaschi, Lara Massai, Matteo Becatti, Alessandra Modesti, Luigi Messori, Tania Gamberi

**Affiliations:** 1 Department of Experimental and Clinical Biomedical Sciences, University of Florence, Viale Morgagni 50, 50134 Firenze, Italy; gorinigiulia@gmail.com (G.G.); francesca.magherini@unifi.it (F.M.); tania.fiaschi@unifi.it (T.F.); matteo.becatti@unifi.it (M.B.); alessandra.modesti@unifi.it (A.M.); 2 Department of Chemistry ‘Ugo Schiff’, University of Florence, via della Lastruccia 3-13, Sesto Fiorentino, 50019 Firenze, Italy; lara.massai@unifi.it

**Keywords:** gold(III)-based compounds, thioredoxin reductase, mitochondria, apoptosis signal pathway, A2780 ovarian cancer cells

## Abstract

Au_2_phen ((2,9-dimethyl-1,10-phenanthroline)_2_Au_2_(µ-O)_2_)(PF_6_)_2_ and Auoxo6 ((6,6′-dimethyl-2,2′-bipyridine)_2_Au_2_(µ-O)_2_)(PF_6_)_2_ are two structurally related gold(III) complexes that were previously reported to display relevant and promising anticancer properties in vitro toward a large number of human cancer cell lines. To expand the knowledge on the molecular mechanisms through which these gold(III) complexes trigger apoptosis in cancer cells, further studies have been performed using A2780 ovarian cancer cells as reference models. For comparative purposes, parallel studies were carried out on the gold(III) complex AuL12 (dibromo(ethylsarcosinedithiocarbamate)gold(III)), whose proapoptotic profile had been earlier characterized in several cancer cell lines. Our results pointed out that all these gold(III) compounds manifest a significant degree of similarity in their cellular and proapoptotic effects; the main observed perturbations consist of potent thioredoxin reductase inhibition, disruption of the cell redox balance, impairment of the mitochondrial membrane potential, and induction of associated metabolic changes. In addition, evidence was gained of the remarkable contribution of ASK1 (apoptosis-signal-regulating kinase-1) and AKT pathways to gold(III)-induced apoptotic signaling. Overall, the observed effects may be traced back to gold(III) reduction and subsequent formation and release of gold(I) species that are able to bind and inhibit several enzymes responsible for the intracellular redox homeostasis, in particular the selenoenzyme thioredoxin reductase.

## 1. Introduction

Over the last few years, numerous studies have highlighted the importance of gold compounds as an interesting class of new anticancer drug candidates characterized by peculiar mechanisms of action [1,2]. In particular, a variety of gold(III) and gold(I) compounds, bearing different structural motifs, were found to produce extensive cell death in several cancer cell lines, with remarkable selectivity for cancer over normal cells [3,4,5]. The modes of action and the targets of antiproliferative gold compounds appear to be multifaceted and distinct from those of platinum compounds (i.e., cisplatin), yet the “true” molecular mechanisms remain largely unknown [6] and require further studies. A number of structurally diverse gold(III) compounds, including both classical coordination complexes and organometallic derivatives, have been investigated in our laboratory as perspective anticancer agents starting from the year 2000 onwards and some initial structure activity relationships have been drawn [7]. A common feature of these compounds is that they are easily reduced to gold(I) complexes in physiological environments. Consequently, their cytotoxic effects have been ascribed both to their strong oxidising character and to coordination preferences and direct protein binding of the resulting gold(I) species [8,9].

**Figure 1:**
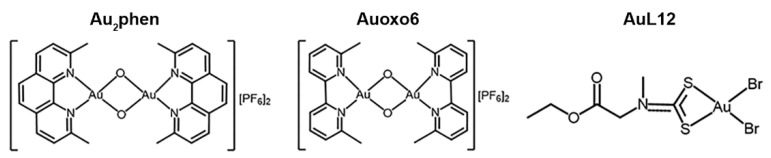
Chemical structure of the investigated gold(III) compounds: Au_2_phen ((2,9-dimethyl-1,10-phenanthroline)_2_Au_2_(µ-O)2)(PF_6_)_2_, Auoxo6 ((6,6′-dimethyl-2,2′-bipyridine)_2_Au_2_(µ-O)_2_)(PF_6_)_2_ and AuL12 (dibromo(ethylsarcosinedithiocarbamate)gold(III)).

Au_2_phen ((2,9-dimethyl-1,10-phenanthroline)_2_Au_2_(µ-O)2)(PF_6_)_2_ and Auoxo6 ((6,6′-dimethyl-2,2′-bipyridine)_2_Au_2_(µ-O)_2_)(PF_6_)_2_ are two representative members of the group of gold(III) compounds that have attracted a lot of attention for their excellent biological profile. They are dioxo-bridged dinuclear gold(III) complexes with two 2,9-dimethylphenanthrolines or bipyridyls as terminal ligands, as depicted in Figure 1.

Auxo6 was first prepared in 1998 [10] and was further characterized in 2006 and 2008 [11,12], while Au_2_phen was first described in 2010 [13]. Remarkably, both compounds revealed acceptable stability under physiological conditions and excellent antiproliferative properties in vitro against a large panel of 36 cancer cell lines, combining a high cytotoxic potency with a pronounced tumor selectivity [3,13]. Gold(III) reduction, dioxo bridge disruption, coordinative gold(I) binding to proteins, and concomitant release of the N-heterocyclic ligand seem to be the key processes occurring in the biological environment, yet the final protein targets are still unknown or only partially known.

Given the promising and interesting features of these compounds, we decided to perform a more extensive investigation on the mechanisms through which these gold(III) complexes trigger cancer cell death. Our study was inspired by a similar study carried out a few years ago on the gold(III) compound AuL12 (dibromo(ethylsarcosinedithiocarbamate)gold(III)), a cytotoxic dithiocarbamate gold(III) complex developed by Fregona in Padua [14], which similarly to Auoxo6 and Au_2_phen, may undergo activation through facile gold(III) into gold (I) reduction. Indeed, although structurally different, the gold(III) complex AuL12 shows strict similarities with the two study complexes in terms of reactivity within a biological environment, as AuL12, similarly to Auoxo6 and Au_2_phen, may undergo facile reduction in the presence of biological reductants, e.g., GSH. In all cases, gold(III) reduction typically results in the loss of the original ligands and in the formation of free gold(I) ions, which may interact directly with proteins selectively at the level of cysteine or histidine residues [9,13,15].

In 2012, Fregona et al. published a detailed paper where the proapoptotic effects of AuL12 were illustrated in different cancer cell lines [16]. This study provided clear evidence that AuL12 induces oxidative stress and tumor cell death by both favoring the opening of the mitochondrial permeability transition pore (MPTP) and activating the proapoptotic protein Bax of the Bcl-2 family.

Taking all these aspects into account, we explored the apoptotic signaling modulated by Au_2_phen and Auoxo6 in A2780 human ovarian cancer cells. In addition, parallel experiments were realized on AuL12 for comparative purposes.

## 2. Materials and Methods

### 2.1. Materials

RPMI 1640 cell culture medium, fetal bovine serum (FBS), and phosphate-buffered saline (PBS) were obtained from Euroclone (Milan, Italy). Thiazolyl blue tetrazolium bromide (MTT) was obtained from Merck Life Science (Milan, Italy). Tetra-methyl-rhodamine methyl ester (TMRM) and 2′,7′-dichlorodihydrofluorescein diacetate (H2DCF-DA) were purchased from Molecular Probes (ThermoFisher Scientific, Waltham, MA, US). Enhanced chemiluminescence (ECL) Western blot detection reagents, non-fat dry milk, 4–20% precast SDS-PAGE, and polyvinylidene difluoride (PVDF) membranes were procured from Bio-Rad Laboratories (Hercules, USA). General chemicals were purchased from Merck Life Science (Milan, Italy), unless otherwise indicated. All chemicals were of analytical or higher grade.

### 2.2. Chemical Synthesis and Drug Treatment

The gold(III) compound Au_2_phen was synthesized and purified as described by Cinellu et al. [13]. The gold(III) compound Auoxo6 was synthesized and purified as described by Casini et al. [11], while the gold(III) compound AuL12 was synthesized and purified as previously described by the research group of Fregona [14].

To carry out the A2780 treatments described throughout the manuscript, the three gold(III) compounds were dissolved in dimethyl sulfoxide (DMSO), as a stock solution (20mM), and the calculated amounts of each drug solution were then diluted in the cell growth medium to reach a final DMSO concentration of 0.1% (*v*/*v*); this concentration of DMSO in A2780 culture has been reported neither to induce apoptosis nor to activate caspases [17]. The same DMSO concentration was added to the control cell medium.

### 2.3. Cell Line and Culture Conditions

To perform the experiments, A2780 human ovarian cancer cell line, purchased from the European Collection of Authenticated Cell Cultures (ECACC, a part of Public Health England) (Lot Nº 13J012, Sigma Aldrich, St. Louis, MO, USA), was used as in vitro model of ovarian cancer. HEK (human embryonic kidney) and PNT1 (human normal prostate epithelium) cells were gifted from Prof. Letizia Taddei, University of Florence, Italy. Cells were maintained in RPMI1640 medium supplemented with 10% FBS, 1% glutamine, and 1% antibiotics at 37 °C and subcultured twice weekly. Split 1:5 (3–6 × 10^4^ cells per mL).

### 2.4. Study of the Cytotoxic Effects on A2780 Cell Line

The inhibition of cell proliferation by Au_2_phen, Auoxo6, and AuL12 on A2780 cell line was evaluated through MTT (3-(4,5-dimethylthiazol-2-yl)-2,5-diphenyltetrazolium bromide) test. Viable cells can convert MTT into formazan, which is coloured and detectable at 595 nm. Exponentially growing cells were seeded in 96 well-microplates at a density of 8 × 10^3^ for 24 h and then gold compounds were added in fresh RPMI medium at concentrations ranging from 0.003 to 100 µM and incubated for 72 h. On the day of the test, cells were treated with 0.5 mg/mL MTT for 1 h at 37 °C. Following precipitation, blue formazan was dissolved in DMSO, and the optical density was read in a microplate reader interfaced with Microplate Manager/PV version 4.0 software (BioRad Laboratories, Hercules, USA). From the absorbance measurements, the half-maximal inhibitory concentration (IC_50_) value of each compound on A2780 cells was calculated using GraphPad Prism software version 6.0 (Graphpad Holdings, LLC, USA). The same protocol was applied on non-tumor cell lines, i.e., HEK and PNT1 cells.

The effects of these 72 h exposure IC_50_ doses on A2780 cell viability were also evaluated using an MTT time course assay at 12, 24, 48, and 72 h of drug exposure. The experimental protocol described above was applied.

### 2.5. Assessment of Apoptosis by Flow Cytometry

The evaluation of cell death was carried out using the TACS annexin V/propidium iodide staining kit (Trevigen, Gaithersburg, MD, USA) according to the manufacturer’s instructions. Exponentially growing cells were seeded in 60 mm plates at a density of 8 × 10^5^ for 24 h and then gold compounds were added in fresh RPMI medium at concentrations corresponding to their 72 h exposure IC_50_ doses. After 72 h, controls and treated cells were trypsinized, washed with PBS buffer, and suspended in the staining solution for 15 min at room temperature in the dark. Cells were then washed and immediately analyzed by FACSCantoII flow cytometer (BD Biosciences, New Jersey, USA). Cells were gated and plotted on dot plots showing the distribution of annexin V-positive cells (early apoptotic cells) and annexin V/propidium iodide-positive cells (late apoptotic cells).

In addition, caspase-3, caspase-8, and caspase-9 activation was assessed in treated and untreated cells. Likewise, cells were trypsinized, washed with PBS, and suspended in FAM-FLICATM caspase solution (FAM-DEVD-FMK caspase FLICA kit, ImmunoChemistry Technologies, MN 55431 USA) for 30 min at 37 °C. Cells were then washed twice with PBS and analyzed using a FACSCantoII flow cytometer (BD Biosciences, New Jersey, USA) [18].

### 2.6. Thioredoxin Reductase Activity Assay

The total thioredoxin reductase (TrxR) activity was measured using a commercial colorimetric assay kit (#CS0170, Sigma Aldrich, St. Louis, MO, USA) based on the reduction of 5,5′-dithiobis (2-nitrobenzoic) acid (DTNB) with NADPH to 5-thio-2 nitrobenzoic acid (TNB) at 412 nm. This kit also contains an inhibitor solution of mammalian thioredoxin reductase. Since several enzymes present in the biological sample can reduce DTNB, the specific inhibitor is used to determine the reduction of DTNB due only to TrxR activity. A2780 cells were treated for 6, 12, and 24 h with gold(III) concentrations corresponding to their 72 h exposure IC_50_ doses. Cells were lysed with RIPA buffer (50 mM Tris-HCl pH 7.0, 1% (*v*/*v*) NP-40, 150 mM NaCl, 2 mM ethylene glycol bis(2-aminoethyl ether)tetra-acetic acid, 100 mM NaF) supplemented with a cocktail of protease inhibitors. The protein concentrations in the cell lysates were determined by Bradford protein assay kit (Bio-Rad Laboratories, Hercules, USA) according to the manufacturer’s instructions and then 30 µg samples of proteins were used for the assay. Results were normalized to the cellular protein content.

### 2.7. Intracellular ROS Evaluation

Intracellular ROS were measured after 24 and 48 h of treatment with 72 h exposure IC_50_ doses using 2′,7′-dichlorodihydrofluorescein diacetate (H2DCF-DA), a cell-permeable fluorogenic dye able to measure cellular hydroxyl, peroxyl, and other ROS. Within the cell, H2DCF-DA is de-acetylated by cellular esterases to a non-fluorescent compound, which becomes fluorescent once it is oxidized by cellular ROS into 2′,7′-dichlorofluorescein (DCF). Cells were trypsinized, washed, and incubated with 5 µM H2DCFDA for 30 min in the dark. Then, cells were washed again and immediately analyzed using a FACSCantoII (BD Biosciences, New Jersey, USA) flow cytometer [19].

### 2.8. Measurement of Mitochondrial Membrane Potential

The mitochondrial membrane potential (Δψm) was measured using tetramethylrhodamine methyl ester perchlorate (TMRM), a lipophilic potentiometric fluorescent dye that distributes between the mitochondria and cytosol in proportion to Δψm by virtue of its positive charge. The amount of accumulated dye in the mitochondria, and consequently the signal intensity, is a direct function of mitochondrial potential. A2780 cells were treated with gold(III) compounds for 24 and 48 h with 72 h exposure IC_50_ doses. Cells were then trypsinized, washed with PBS, and incubated with 100 nM TMRM for 20 min in the dark at 37 °C. After labelling, cells were washed again, suspended in PBS, and analyzed using a FACSCantoII flow cytometer (BD Biosciences, New Jersey, USA) [20].

### 2.9. Mitochondrial Respiration Analysis

The analysis of the oxygen consumption rate (OCR) was performed using a Clark-type O_2_ electrode inserted in a thermostated airtight chamber (Oxygraph Plus System, Hansatech Instruments, King’s Lynn, Norfolk, UK). Briefly, control and treated cells (12 and 24 h) were trypsinized, washed with PBS, and suspended in culture medium to measure the steady state OCR for 10 min. The OCR values, expressed as nmol of consumed O_2_/mL/min, were normalized to the cellular protein content [21]. To evaluate the response of complex I, complex II/III, and ATP-synthase-driven OCRs in the presence or absence of gold-treatment, we used the protocol described by Baracca et al. [22]. Cells were trypsinized, washed, and resuspended in buffer containing sucrose, TRIS-HCl, MgSO_4_, EDTA, and KH_2_PO_4_. Cell suspension was treated with digitonin to allow cell membrane permeabilization. To stimulate complex I, 250 mM glutamate/malate was added, whereas to stimulate complex II, 250 mM succinate was added (state 2 respiration). Then, to induce ATP-synthase-driven mitochondrial respiration, 10 mM ADP was added (state 3 respiration). To estimate the mitochondrial respiratory capacity, defined as the respiratory control ratio (RCR), state 3/state 2 ratios were calculated for control and treated cells.

### 2.10. Lactate Production Assay

The lactate amount was measured in cell culture media after 24 h of treatment with K-LATE kit (Megazyme, Bray, Ireland) according to the data sheet provided by the manufacturer. Lactate content was normalized on the protein content of the same sample.

### 2.11. Western Blot Analysis

After drug treatments, cells were lysed in RIPA buffer supplemented with a cocktail of protease and phosphatase inhibitors. The protein concentrations in the cell lysates were determined by Bradford protein assay kit (Bio-Rad Laboratories, Hercules, USA) according to the manufacturer’s instructions. To favor the electrophoretic separation, 25% sample loading buffer (SLB) was added to the cell lysates. Here, 20 µg samples of proteins from controls and treated cells were loaded on 4–20% pre-cast SDS-PAGE gels and blotted onto PVDF membranes. Primary antibodies Bcl-2 (1:1000 dilution,#sc-7382, Santa Cruz, Dallas, Texas USA), Bax (1:1000 dilution, #sc-493, Santa Cruz), OXPHOS cocktail (1:1000 dilution, #ab110413, AbCam, Cambridge, UK), citrate synthase (1:1000 dilution, #sc-515640, Santa Cruz), ASK1 (1:1000 dilution, #sc-390275, Santa Cruz), phospho(Thr838)-ASK1 (1:1000 dilution, #CPA6042, Cohesion Biosciences, London, UK), AKT (1:1000 dilution, #sc-5298, Santa Cruz), and phospho(Ser473)-AKT (1:1000 dilution, #GTX128414, GeneTex, Irvine, CA, USA) were added to 2% non-fat dry milk in PBS-tween solution, while PVDF membranes were incubated overnight at 4 °C. Then, membranes were treated with horseradish peroxidase (HRP)-conjugated secondary antibodies (1:5000 dilution, Santa Cruz) and immunoreactive bands were detected with an ECL kit detection system on an Amersham Imager 600 (GE Healthcare, Chicago, IL, USA). For the quantification, a densitometric analysis of the bands was performed using ImageJ2 software version 2.0.0-rc-64 [23]. The intensities of the immunostained bands were normalized on the Coomassie brilliant blue R-250-stained total protein from the same PVDF membrane.

### 2.12. Statistical Analysis

All values are given as means ±SD of no less than three independent experiments. Statistical analysis was performed by one-way ANOVA test followed by Tukey’s multiple comparison test using GraphPad Prism software version 6.0 (Graphpad Holdings, LLC, USA). A *p*-value ≤ 0.05 was considered statistically significant. For details, see figure legends.

## 3. Results

### 3.1. Antiproliferative Activity of Au_2_phen, Auoxo6, AuL12

The antiproliferative effects of each gold(III) compound were assayed as measurements of the cells’ metabolic activity via MTT (3,(4,5-dimethylthiazol-2)2,5 difeniltetrazolium bromide) test. The representative dose–response results are shown in Figure S1 of the Supplementary Materials. Table 1 reports the IC_50_ values for each compound obtained at 72 h on A2780 human ovarian carcinoma cells and non-tumor PNT1 and HEK cells.

It was observed that Au_2_phen and Auoxo6 displayed significant antiproliferative effects on A2780 cells with IC_50_ values falling in the micromolar range: 0.8 ± 0.02 µM for Au_2_phen, 1.14 ± 0.18 µM for Auoxo6, and 4.00 ± 0.03 µM for AuL12. Interestingly, IC_50_ values of Au_2_phen and Auoxo6 were respectively about 14-fold and 16-fold higher for PNT1 and similarly 5-fold higher for HEK cells than those obtained for the A2780 cell line. In the case of AuL12-treated cells, IC_50_ values were 3-fold higher for PNT1 cells and 2.7-fold higher for HEK cells compared to those found for A2780 cells. Additionally, from the observation of the IC_50_ values obtained on the A2780 cells, the cytotoxic potency of Au_2_phen and Auoxo6 was higher than that of AuL12 (i.e., 5- and 3.5-fold, respectively).

### 3.2. Apoptotic Profile Analysis and Caspase Activation

**Figure 2:**
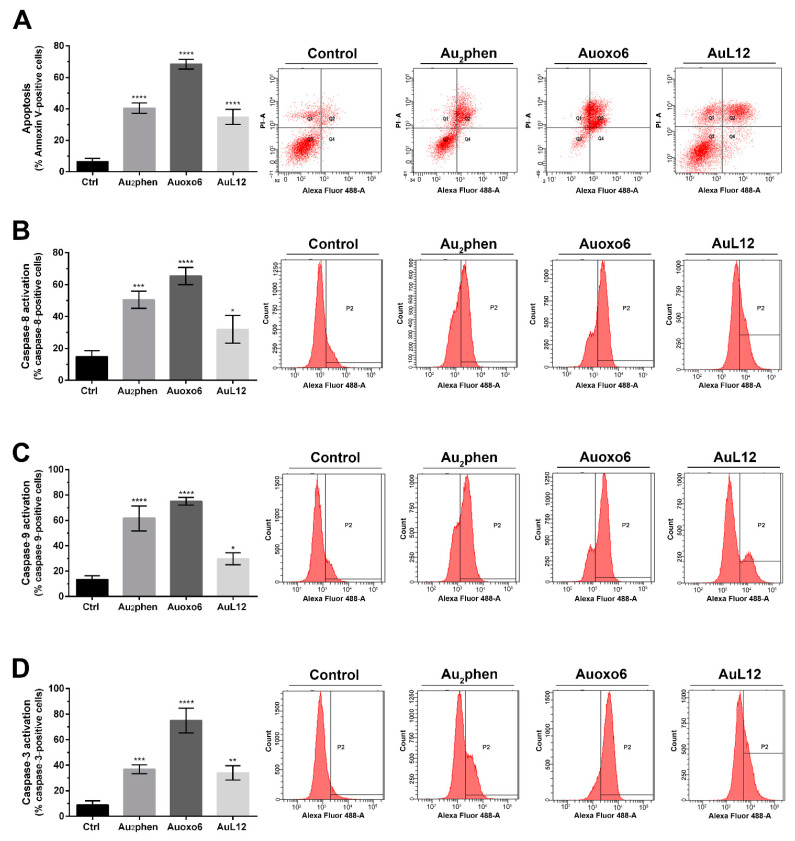
Apoptotic profile analysis and caspases activation: (**A**) percentages of apoptotic cells shown by flow cytometry analysis of annexin V/propidium iodide-stained A2780 cells treated for 72 h with Au_2_phen, Auoxo6, and AuL12 IC_50_ doses; (**B**) caspase-8, (**C**) caspase-9, and (**D**) caspase-3 activity shown by fluorescence-activated cell sorting analysis using FAM FLICA caspase assay kits in A2780 cells treated for 72 h with gold(III) complex concentrations corresponding to their 72 h IC_50_ dose. Flow cytometric images are representative of three independent experiments. Histograms report the mean values ±SD. The statistical analysis was carried out using one-way ANOVA test followed by Tukey’s multiple comparisons test using GraphPad Prism software v 6.0 (* *p* < 0.05, ** *p* < 0.01, *** *p* < 0.001, **** *p* < 0.0001).

The apoptotic profile was checked through flow cytometry using the annexin V/propidium iodide staining on controls and Au_2_phen-, Auoxo6-, and AuL12-treated cells upon 72 h exposure at their IC_50_ dose. Figure 2A shows the histograms reporting the mean values of the percentage of annexin V-positive cells from three independent experiments, along with representative flow cytometric dot plots.

The obtained results pointed out that Au_2_phen, Auoxo6, and AuL12 significantly increased the annexin V fluorescence intensity (by 6-, 10-, and 5-fold, respectively) in comparison with untreated control cells, indicating that these gold-based complexes favor apoptosis as the preferred mode of cell death.

The analysis continued by testing whether the apoptotic process observed through annexin V/propidium iodide test was activated using the intrinsic–cytosolic or extrinsic–mitochondrial pathway. To shed light on this issue, we assayed caspase activation through FACS analysis in control and cells exposed to the drugs for 72 h. Caspase-8 (extrinsic pathway) and caspase-9 (intrinsic pathway) were found to be more significantly activated in Au_2_phen-, Auoxo6-, and AuL12-treated cells compared to controls (Figure 2B). Regarding caspase-8 activation, around 50% of Au_2_phen-treated cells and 70% of Auoxo6-treated cells showed significant activation compared to controls. Instead, lower caspase-8 activation was observed in AuL12-treated cells, even though it was significant (~30%) (Figure 2B). Likewise, 60% of Au_2_phen-treated cells and 75% of Auoxo6-treated cells showed significantly higher caspase-9 activation compared to control cells, whilst lower variation was observed in AuL12-treated cells (~30%) (Figure 2C). In accordance with the results regarding caspase-8 and caspase-9 activation, the fluorescence intensity derived from caspase-3 activation was significantly higher in Auoxo6-treated cells when compared to controls (about 80%), while a minor but significant change was detected upon Au_2_phen and AuL12 exposure (~40%) (Figure 2D).

### 3.3. Effects on Thioredoxin Reductase Activity and Cell Redox Balance

The thioredoxin reductase (TrxR)–thioredoxin (Trx) redox system has already been identified as the major target of gold-based complexes [24,25,26,27,28,29,30], including AuL12 [31]; therefore, we decided to evaluate whether the proposed mechanism could be extended to Au_2_phen and Auoxo6 in the A2780 cell line. For this aim, we assessed the effects of Au_2_phen and Auoxo6 compounds in comparison to AuL12 after 6, 12, and 24 h exposure, both on the activity of the TrxR enzyme and cell redox balance. At these incubation times, treated cells were viable, as demonstrated by the MTT time course experiments shown in Figure S2; therefore, these treatment conditions allowed us to analyze whether the inhibition of the TrxR–Trx system was an early cellular event that could lead to apoptosis without causing cell death.

**Figure 3:**
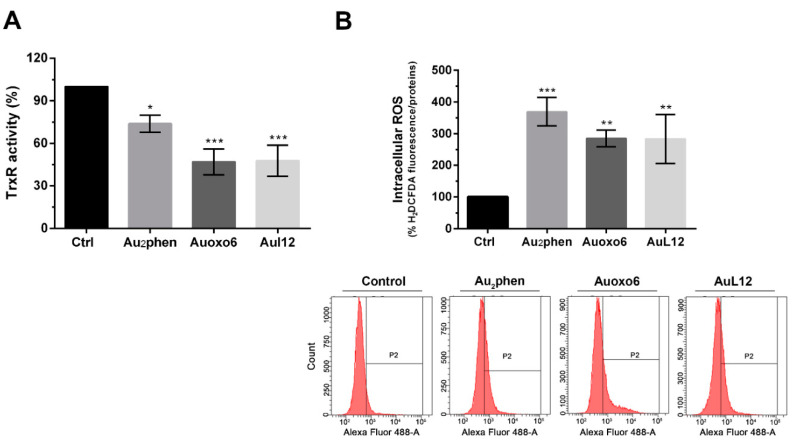
Effects on thioredoxin reductase activity and cell redox balance. A2780 cells were treated with Au_2_phen, Auoxo6, and AuL12 72 h IC_50_ doses: (**A**) TrxR enzyme inhibition assay was performed after 24 h of treatment using a commercial thioredoxin reductase assay kit, whereby histograms report the percentages of TrxR enzyme activity on gold(III)-treated cells in comparison with controls; (**B**) intracellular ROS production was evaluated after 48 h of treatment through the detection of fluorogenic H_2_DCFDA dye using a flow cytometer, whereby histograms report the percentages of fluorescent treated cells compared to controls. Flow cytometric images are representative of three independent experiments. All experiments were performed in triplicate. The statistical analysis was carried out using one-way ANOVA test followed by Tukey’s multiple comparisons test using Graphpad Prism software v 6.0 (* *p* < 0.05, ** *p* < 0.01, *** *p* < 0.001).

The TrxR enzyme activity was quantified through the measurement of the colorimetric reduction of DTNB into TNB at 412 nm. The results pointed out that upon 6 h exposure, the enzyme activity levels were similar in controls and treated cells, whereas after 12 h, Au_2_phen and Auoxo6, as with AuL12, showed a clear tendency to inhibit the TrxR activity, even if the changes were not statistically significant. Following a 24 h treatment, Auoxo6 and AuL12 were proved to markedly inhibit (about 50%) TrxR enzyme activity, which on the other hand was much less affected by Au_2_phen (~26%) (Figure 3A).

To verify whether this impairment of TrxR activity was able to impact the redox homeostasis within cells, we assayed ROS levels in controls and Au_2_phen-, Auoxo6-, AuL12-treated A2780 cells for 24 and 48 h. The fluorogenic dye 2′,7′-dichlorfluorescein-diacetate (H_2_DCFDA) was used to mark intracellular ROS, while the fluorescence intensities were analyzed using flow cytometry. Upon 24 h treatment, no statistically significant differences could be observed between controls and treated cells. On the contrary, as shown in Figure 3B, all gold(III) compounds elicited significant oxidative stress after 48 h treatment. Au_2_phen-treated cells were affected by a consistent enhancement of ROS (about 3.7-fold higher than control cells), whereas Auoxo6 and AuL12 showed slightly lower increases of ROS (about 2.8-fold higher than controls).

### 3.4. Involvement of Mitochondrial Membrane Potential Impairment in Gold(III)-Induced Apoptosis

**Figure 4:**
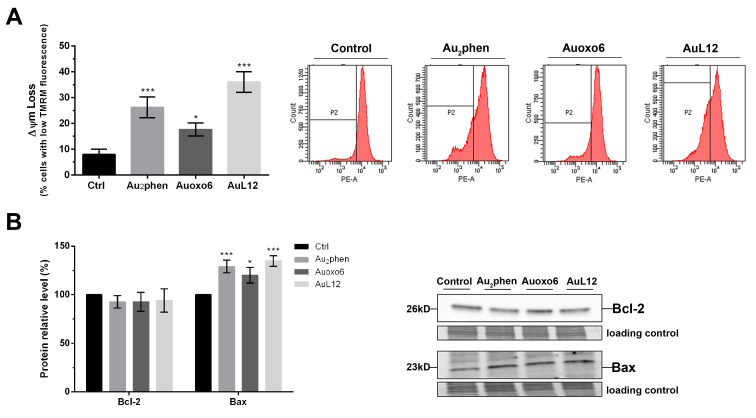
Involvement of mitochondrial membrane potential (Δψm) dysfunction in gold(III)-induced apoptosis. A2780 cells were treated for 48 h with Au_2_phen, Auoxo6, and AuL12 72 h IC_50_-doses: (**A**) Δψm values obtained using fluorescent TMRM dye and flow cytometry analysis, whereby flow cytometric images are representative of three independent experiments and histograms report the percentages of treated cells with low TMRM fluorescence compared to controls; (**B**) Western blot analysis of Bcl-2 and Bax contents, whereby histograms report the normalized mean relative integrated density (±SD) values of Bcl-2 and Bax immunostained bands compared to controls. Representative immunoblots are also shown together with the matching Coomassie-stained PVDF membranes, which were used as loading control. Original Western blot images of Bcl-2 and Bax content are reported in Figure S3. All experiments were performed in triplicate. The statistical analysis was carried out using one-way ANOVA test followed by Tukey’s multiple comparisons test using GraphPad Prism software v 6.0 (* *p* < 0.05, *** *p* < 0.001).

As suggested by the literature, the inhibition of the thioredoxin reductase–thioredoxin redox system by gold(I) and gold(III) complexes (e.g., auranofin and AuL12) and the subsequent production of ROS are able to trigger cancer cell apoptosis through mitochondrial impairment [16,32,33,34,35,36]. Indeed, alterations of the mitochondrial membrane potential (Δψm) and permeability are crucial steps in the apoptotic process, allowing the release of proapoptotic factors into the cytoplasm [37]; hence, to assess whether the gold(III) compounds Au_2_phen and Auoxo6 would affect mitochondrial functions in a similar way to AuL12, we examined Δψm changes in A2780-treated cells using tetramethylrhodamine methyl ester (TMRM), a cell-permeant, cationic red fluorescent dye that specifically accumulates in negatively charge polarized mitochondria. In line with ROS detection, upon 24 h treatment, no statistically significant differences could be detected between controls and treated cells; however, after 48 h exposure, all of the selected gold(III) compounds successfully led to loss of Δψm. As shown in Figure 4A, Au_2_phen and AuL12 significantly increased the cells bearing lower Δψm values (by about 26% and 36%, respectively), whereas Auoxo6 induced a Δψm decrease only in 17% of cells.

It has been reported that Bcl-2 family members are involved in the control of mitochondrial membrane integrity and permeability. Since the imbalance of proapoptotic and antiapoptotic Bcl-2 family proteins may lead to the loss of mitochondrial membrane potential and promote the activation of apoptotic pathways [38], we wondered whether the selected drugs could affect the expression levels of prosurvival Bcl-2 and proapoptotic Bax, two members of Bcl-2 family; therefore, we performed a Western blot analysis on A2780 cells exposed to 24 and 48 h treatment. In line with TMRM experiments, the 24 h exposure did not modify Bcl-2 or Bax protein levels. On the other hand, after 48 h, we detected significant increases of proapoptotic Bax (about 29% upon Au_2_phen, 20% upon Auoxo6, and 34% upon AuL12 treatments, all compared to controls). With regard to antiapoptotic Bcl-2, all gold(III) complexes showed a tendency to decrease without reaching statistically significant values (Figure 4B).

### 3.5. Metabolic Changes Elicited by Gold(III) Compounds

**Figure 5:**
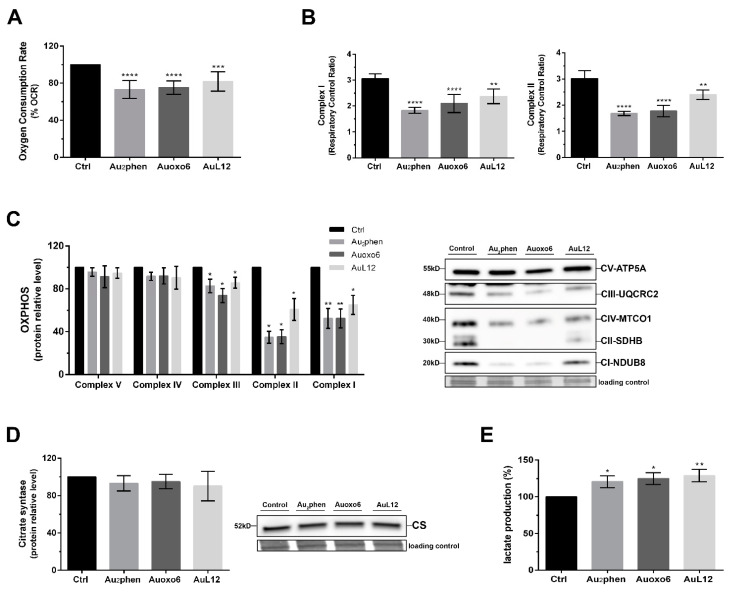
Influence of gold(III) complexes on mitochondrial metabolism. A2780 cells were treated for 24 h with Au_2_phen, Auoxo6, and AuL12 72 h IC_50_ doses: (**A**) The percentage of oxygen consumption rate (OCR) and (**B**) the respiratory control ratio (RCR) values were measured using a Clark-type O_2_ electrode from Hansatech Instruments (King’s Lynn, Norfolk, UK) as described in Materials and Methods. The protein amount of (**C**) oxidative phosphorylation (OXPHOS) complexes and (**D**) citrate synthase (CS) was evaluated using Western blot analysis. Histograms report the normalized mean relative integrated density (±SD) values for each OXPHOS complex, and CS immune-stained bands compared to controls. Representative immunoblots are also shown together with the matching Coomassie-stained PVDF membranes, which were used as loading control. Original Western blot images of OXPHOS and CS content are reported in Figures S4 and S5. (**E**) The lactate amounts were detected in 1 mL of supernatant medium using a commercial L-lactic acid assay kit (Megazyme, Bray, Ireland). Histograms report the percentages of mean values ±SD with respect to controls. All experiments were performed in triplicate. The statistical analysis was carried out using one-way ANOVA test followed by Tukey’s multiple comparisons test using GraphPad Prism software v 6.0 (* *p* < 0.05, ** *p* < 0.01, *** *p* < 0.001, **** *p* < 0.0001).

To obtain a more detailed overview of the involvement of mitochondria in apoptotic cell death induced by gold(III) complexes, we investigated their effects on mitochondrial metabolism; hence, we analyzed whether the observed decrease of Δψm might be associated with a concomitant decrease in mitochondrial respiration efficiency. As a first step, we measured the mitochondrial oxygen consumption rate (OCR) in controls and 24-h-treated A2780 cells using a Clark-type electrode inserted into a thermostated airtight chamber, as described in Materials and Methods. As depicted in Figure 5A, all gold(III) compounds elicited decreases in OCR values compared to controls. Higher reduction levels were achieved with Au_2_phen (26.8%) and Auoxo6 (24.7%), whereas in AuL12-treated cells, the reduction level was slightly lower (18%).

Afterwards, to confirm the effects of gold(III) compounds on mitochondrial respiration, we assessed the mitochondrial electron transport chain (ETC) efficiency in permeabilized A2780 cells with the same exposure time. Cell membranes were permeabilized with digitonin to obtain intact mitochondria suspensions. First, saturating glutamate–malate or succinate substrates were used to start complex-I- or complex-II-driven respiration, respectively (referred to as “state 2 respiration”). Then, saturating ADP was added to stimulate ATP-synthase-driven respiration (referred to as “state 3 respiration”). The ratio of the “state 3 respiration” to the “state 2 respiration” is defined as the respiratory control ratio (RCR) and is a measurement of the oxidative phosphorylation (OXPHOS) efficiency. As shown in Figure 5B, when mitochondrial respiration was driven by complex I, all gold(III) compounds showed lower RCR values compared to controls. Indeed, the RCR mean value for Au_2_phen was about 1.8, for Auoxo6 was 2.1, and for AuL12 was 2.3, whereas for control cells the RCR was about 3.1. Likewise, when mitochondrial respiration was driven by complex II, significantly lower RCR values were found for Au_2_phen (about 1.7), Auoxo6 (about 1.8), and AuL12 (about 2.4)-treated cells compared to those obtained in controls (about 3.0) (Figure 5B). Following OCR analysis, we explored OXPHOS protein levels to understand whether the differences observed in RCR could relate to alterations in the content of the ETC complexes. A2780 cells were treated with Au_2_phen, Auoxo6, and AuL12 at their 72 h IC_50_ doses for 24 h, then subsequently analyzed through Western blotting. As shown in Figure 5C, none of the drugs significantly changed the complex V content. On the contrary, complex I, II, III, and IV levels changed upon treatment with each gold compound. In detail, all drugs triggered similar decreases of complex III and IV, whereas stronger effects on both complex I and II levels were achieved upon Au_2_phen and Auoxo6 treatment. Indeed, Au_2_phen led to decreases of 48% for complex I and of 66% for complex II. Likewise, Auoxo6 induced a 50% reduction of complex I and a 70% reduction of complex II. Regarding AuL12, a 28% decrease of complex I and a 40% decrease of complex II were observed in comparison with controls.

Furthermore, to verify whether the impairment of mitochondrial respiration efficiency could be due to a loss in the mitochondrial content, we analyzed the amount of mitochondrial citrate synthase (CS), the rate-limiting enzyme of TCA cycle, which is commonly used as an indicator of the mitochondrial mass. Cells were treated for 24 h with Au_2_phen, Auoxo6, and AuL12 at their 72 h IC_50_ doses, then cell lysates were analyzed through Western blotting. As reported in Figure 5D, none of the drugs caused variations in CS levels. These results confirmed the ability of gold(III) compounds to impair the mitochondrial proton electrochemical gradient; therefore, we tested whether such mitochondrial damage could lead to the metabolic shift towards glycolysis. To evaluate the possible enhancement of glycolysis, we measured the lactate amounts contained in the media of both control and 24-h-treated cells. As depicted in Figure 5E, all treatments significantly enhanced lactate levels. Indeed, we reported increases of about 20% with Au_2_phen, 25% with Auoxo6, and 29% with AuL12.

### 3.6. Contribution of ASK1 and AKT in Gold(III)-Induced Apoptotic Signaling

**Figure 6:**
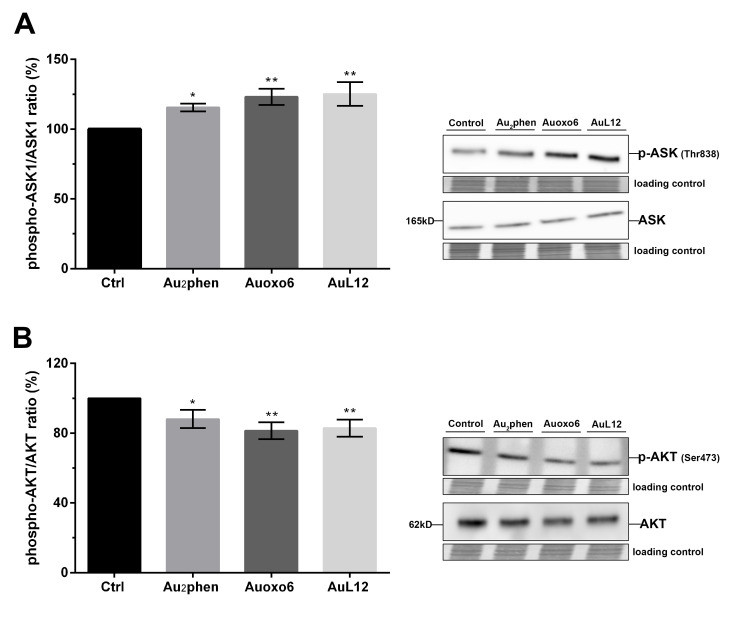
Contributions of ASK1 and AKT in gold(III)-induced apoptotic signaling. A2780 cells were treated for 24 h with Au_2_phen, Auoxo6, and AuL12 72 h IC_50_ doses. The phosphorylation levels and total protein amounts of (**A**) ASK1 and (**B**) AKT were analyzed through Western blotting. Histograms report the percentages of normalized mean values ± SD of the phospho-ASK1/total ASK1 ratio and phospho-AKT/total AKT ratio compared to controls. The phospho-ASK1/total ASK1 and phospho-AKT/total AKT ratios were obtained from the normalized relative integrated density values of immunostained bands of three independent Western blots. Representative immunoblots are also shown together with the matching Coomassie-stained PVDF membranes, which were used as loading control. Original Western blot images of ASK1 and AKT phosphorylation level and of their total amount are reported in Figures S6 and S7. The statistical analysis was carried out using one-way ANOVA test followed by Tukey’s multiple comparisons test using GraphPad Prism software v 6.0 (* *p* < 0.05, ** *p* < 0.01).

The TrxR–Trx system regulates the redox state and the activity of key proteins involved in cell proliferation and survival [39,40]. Indeed, the Trx-reduced form associates with ASK1, preventing apoptotic cell death. Conversely, Trx oxidation triggers ASK1 dissociation, which then stimulates the apoptotic pathway leading to cell death [41,42,43,44]; hence, to demonstrate the possible contribution of ASK1 in gold(III)-induced apoptosis, we examined its activation in cells treated for 24 h with Au_2_phen, Auoxo6, and AuL12 by Western blot analysis of its phosphorylation levels on Thr 838 residue. The results pointed out that Au_2_phen led to about 15% enhancement of phospho-ASK1 levels (phosphorylated-ASK1/total ASK1 ratio), Auoxo6 led to about 23% enhancement, and AuL12 led to about 25% enhancement, whereas the level of total ASK1 was not affected by any treatment (Figure 6A).

Trx has been known to also affect protein kinase pathways regulating cell proliferation and survival. Among these, the PI3K/AKT pathway has been demonstrated to be inhibited by auranofin [40]. In order to investigate the possible role of this pathway in gold(III)-induced apoptosis, we examined both AKT protein amounts and the phosphorylation state of its Ser 473 in cells treated for 24 h with gold(III) compounds using Western blot analysis. As shown in Figure 6B, all drugs significantly triggered decreases of phospho-AKT levels, whereas the expression levels of total protein were not statistically affected. In detail, the reduction (phosphorylated-AKT/total AKT ratio) rates were of about 12% with Au_2_phen, 19% with Auoxo6, and 17% with AuL12.

## 4. Discussion

In the present study, we intended to analyze the apoptotic signaling induced by the selected gold(III) compounds Au_2_phen and Auoxo6 on the human A2780 ovarian carcinoma cell line in comparison with that triggered by the well-known AuL12. Both Au_2_phen and Auoxo6 revealed remarkable antiproliferative activity levels in vitro towards several human cancer cell lines, showing the ability to induce apoptotic cell death [3,4,11,13,14,33,45,46]. Additionally, with regard to AuL12, a behaviour similar to that of certain gold(I) compounds such as auranofin was demonstrated. In fact, it was shown that AuL12 is able to inhibit thioredoxin reductase activity leading to mitochondrial membrane potential damage, mitochondrial swelling, and finally apoptotic cell death [16,31]. Likewise, Au_2_phen and Auoxo6 turned out to be inhibitors of TrxR in vitro [8], suggesting similarities with AuL12 and gold(I) compounds in activating apoptotic cell death. These findings prompted us to carry out further investigations aimed at elucidating whether the key events for the cytotoxic and proapoptotic effects of these gold(III) compounds could be the alterations of the intracellular redox state and mitochondrial dysfunction.

### 4.1. Antiproliferative and Proapoptotic Effects

Within this framework, we started by comparing the antiproliferative effects of Au_2_phen, Auoxo6, and AuL12 in A2780 cancer cells and in non-tumor PNT1 and HEK cells and confirmed the potential anticancer activity levels of all drugs. Indeed, they all displayed IC_50_ values lying within the micromolar and submicromolar ranges on A2780 ovarian cancer cells, while the non-malignant PNT1 and HEK cells were affected only at higher concentrations. Notably, Au_2_phen and Auoxo6 turned out to be more effective than AuL12 in reducing the viability of A2780 cancer cells. In line with other gold(I) and gold(III) compounds [24,30,46,47,48], the analysis of the apoptotic profile revealed that all of the examined gold compounds favor apoptosis as the main mode of cell death, albeit with different degrees of effectiveness. Indeed, after 72 h treatment, Auoxo6 displayed a higher percentage of apoptotic cells compared to Au_2_phen and AuL12. Likewise, the analysis of caspase cascade activation showed that Au_2_phen and Auoxo6 turned out to be the stronger activators of both caspase-8 (extrinsic pathway) and caspase-9 (intrinsic pathway) initiators as compared to AuL12. Regarding the caspase-3 effector, Au_2_phen and AuL12 displayed similar behavior, revealing slight activation of this pathway compared to Auoxo6. Overall, these results clearly indicate that both extrinsic and intrinsic apoptosis are triggered, although with different degrees of intensity depending on the drug administered.

### 4.2. TrxR Inhibition and ROS Unbalance

The proapoptotic effects of Au_2_phen and Auoxo6 were consistent with the concept developed for auranofin, namely that targeting TrxR would lead to apoptosis via both extrinsic and intrinsic apoptotic pathways [47]. Likewise, Saggioro et al. proposed the impairment of the thioredoxin reductase–thioredoxin redox system by AuL12 as the main mechanism responsible for apoptotic cell death [31]. Subsequently, Chiara et al. provided further evidence that AuL12 exerts anticancer activity by targeting the intracellular redox balance and mitochondria function [16]. In the current study, we confirmed the ability of all three gold(III)-based compounds to inhibit TrxR in A2780 cells after 24 h treatment. Remarkably, Auoxo6 and AuL12 manifested higher inhibitory properties compared to Au_2_phen. These results prompted us to verify whether such TrxR inhibition could trigger oxidative stress, and in fact we found increases in ROS levels after 48 h treatment with all compounds. Unexpectedly, Au_2_phen was the most effective in inducing oxidative stress, which was in contrast to the data obtained from the TrxR inhibition assay. This discrepancy between the TrxR inhibitory capability and ROS increase led us to hypothesize that Au_2_phen could induce cell death not only by impairing the thioredoxin reductase–thioredoxin redox system, but also by exploiting other mechanisms.

### 4.3. Impairment of Mitochondrial Functions

The early inhibition of TrxR activity and the consequent activation of intrinsic apoptotic pathways elicited by Au_2_phen and Auoxo6 prompted us to evaluate whether mitochondria could be a suitable target for their cytotoxicity, as proven for AuL12, gold(III), and gold(I) [31,33]. Indeed, Rigobello et al. [24] provided evidence that auranofin, gold(I), and gold(III) compounds induced the mitochondrial permeability transition and consequent cell death through the inhibition of the mitochondrial thioredoxin reductase. Similarly, AuL12 was proven to alter the mitochondrial membrane potential and permeability in HeLa cells [31]. These effects on mitochondrial functionality were confirmed in the study by Chiara et al. [16]. They showed the ability of AuL12 to inhibit the respiratory complex I, which in turn leads to oxidative stress induction and loss of the mitochondrial membrane potential, favoring cell death. Within this framework, we demonstrated that Au_2_phen and Auoxo6 caused alterations of the mitochondrial membrane potential, even if this occurred with different levels of intensity. Indeed, loss of Δψm was higher for AuL12 and Au_2_phen compared to Auoxo6. Regarding the involvement of mitochondrial dysfunction in AuL12-induced apoptosis, previous studies reported the contributions of the Bcl-2 family members in controlling the mitochondria membrane integrity and permeability [16]. AuL12 was proven to induce cell death by triggering oxidative stress, causing alterations of the mitochondrial membrane potential and permeability, and activating the proapoptotic protein Bax of the Bcl-2 family [16]. Accordingly, we confirmed the increase of proapoptotic protein Bax over the antiapoptotic protein Bcl-2 in AuL12-treated cells. Similarly, and in line with the above reported loss of Δψm, Au_2_phen and Auoxo6 led to increases of Bax, strengthening the hypothesis of mitochondrial involvement in gold(III)-induced cell death. In addition to the impairment of the mitochondrial membrane potential, the investigated gold(III) compounds were proven to alter the mitochondrial metabolism, eliciting reductions of cellular respiratory capacity. Indeed, all three gold(III) compounds triggered decreases of both the oxygen consumption rate and oxidative phosphorylation efficiency, mainly due to an inhibitory effect on respiratory complexes I and II. This respiratory dysfunction was coupled with decreases of OXPHOS complexes I, II, III, and IV; however, such decreases were not linked to reductions in the mitochondrial number. These results allowed us to confirm the previous data reported by Chiara et al. on AuL12 [16] and to highlight the stronger effects of Au_2_phen and Auoxo6 on OXPHOS efficiency. Moreover, this reduced mitochondrial respiratory ability was counteracted by an increase of lactate production, suggesting a metabolic shift towards glycolysis, probably as a compensatory mechanism. Overall, these findings provide further evidence of the ability of the three investigated gold(III) compounds to target mitochondria, impairing their functionalities.

### 4.4. Involvement of ASK1-p38MAPK and PI3K/AKT Signal Pathways

Finally, to provide further evidence on the role of TrxR in gold(III)-induced apoptosis, we evaluated the effects of the examined compounds on two cell survival signaling pathways known to be regulated by the TrxR–Trx system, i.e., ASK1-p38MAPK and phosphoinositide 3-kinase (PI3K)/AKT [39,40]. The TrxR–Trx system plays a central role in controlling the apoptotic pathway, since Trx is a negative regulator of ASK1, a member of the MAPK kinase family. In the reduced form, Trx binds to ASK1, inhibiting its kinase activity, while under oxidative stress the oxidized form of Trx releases ASK1, which in turn activates apoptosis via the JNK and p38MAPK pathways [26,40,48,49]. In the present study, we showed that Au_2_phen, Auoxo6, and AuL12 elicited the activation of ASK1, suggesting the involvement of the Trx–ASK1–p38MAPK signal cascade in gold(III)-mediated cell death. This finding was in line with a previous study by Xinlai Cheng et al. on selected gold(I) N-heterocyclic carbene compounds [50], which demonstrated that the main mechanism of gold(I) carbene compound-mediated cell death was the impairment of the TrxR–Trx system, which in turn leads to the activation of the ASK1-p38MAPK pathway. This pathway was also proven to be essential for the proapoptotic effects of auranofin in promyelocytic leukemia HL-60 cells [47].

Additionally, Trx has been found to activate the PI3K/AKT pathway [40], which in turn promotes cell proliferation, migration, and survival by phosphorylating and inactivating several key apoptotic molecules such as Bad, procaspase-9, and FKHR1(forkhead box protein 1) [51]. Recently, it was reported that auranofin alone or in combination with other drugs targeting TrxR can induce apoptosis through inhibition of the AKT pathway [29,52,53,54,55]. Similarly, the AKT pathway has been proven to take part in the apoptotic cell death induced by the auranofin-related compound chloro(triethylphosphine)gold(I) [56] and an N-heterocyclic carbene–gold(I) complex [57]. In accordance with this evidence, we demonstrated that Au_2_phen, Auoxo6, and AuL12 possess the ability to downregulate the phosphorylation of AKT, thereby suggesting an important role of the AKT pathway in regulating gold(III)-induced cell death.

## 5. Conclusions

In summary, according to the data described above, it is reasonable to propose that TrxR inhibition, which is associated with oxidative stress and mitochondrial dysfunction, is likely to be the dominant mechanism underlying gold(III)-induced apoptosis in human ovarian A2780 cancer cells. Moreover, the signaling pathways ASK1-p38MAPK and PI3K/AKT turned out to be involved in the proapoptotic effects of Au_2_phen, Auoxo6, and AuL12. We can, therefore, state that the similar apoptotic profile shared by both Au_2_phen and Auoxo6 is in agreement with their pronounced structural analogy [13].

## Figures and Tables

**Table 1:** Half-maximal inhibitory concentration (IC_50_) values of the investigated gold compounds after 72 h of treatment using MTT assay.

Compound (µM) ± SD ^1^	Cell Lines		
	A2780	PNT1	HEK293
Au_2_phen	0.80 ± 0.02 µM	11.80 ± 0.25 µM	4.18 ± 0.05 µM
Auoxo6	1.14 ± 0.18 µM	18.40 ± 0.30 µM	6.16 ± 0.04 µM
AuL12	4.00 ± 0.03 µM	12.30 ± 0.17 µM	10.70 ± 0.36 µM

^1^ Values are means ± standard deviation (SD) of three independent experiments.

## Supplementary Materials

The following are available online at https://www.mdpi.com/article/10.3390/biomedicines9080871/s1. Figure S1: Dose–response curves determined using MTT assay. Figure S2: Cell viability time course assay upon gold compound treatment. Figure S3: Original Western blot images of Bcl-2 and Bax contents. Figure S4: Original Western blot images of OXPHOS content. Figure S5: Original Western blot images of citrate synthase (CS) content. Figure S6: Original Western blot images of phospho-ASK1 and total ASK1 contents. Figure S7: Original Western blot images of phospho-AKT and total AKT contents.
