# Supplementary material for: Au2phen and Auoxo6, Two Dinuclear Oxo-Bridged Gold(III) Compounds, Induce Apoptotic Signaling in Human Ovarian A2780 Cancer Cells
Source: Biomedicines. 2021 Jul 23;9(8):871. doi: 10.3390/biomedicines9080871 (PMC8389655; doi:10.3390/biomedicines9080871)
Supplement: Supplementary file 1 [file biomedicines-09-00871-s001.zip › biomedicines-1261126-supplementary.pdf]

Supporting Information

# Au2phen and Auoxo6, two structurally related medicinal gold(III) compounds induce apoptotic signaling in human ovarian A2780 cancer cells

Giulia Gorini [1], Francesca Magherini [1], Tania Fiaschi [1], Lara Massai [2], Matteo Becatti [1], Alessandra Modesti [1], Luigi Messori [2,*] and Tania Gamberi [1,*]

[1]Department of Experimental and Clinical Biomedical Sciences, University of Florence, viale Morgagni 50, Firenze, Italy.; gorinigiulia@gmail.com (G.G.); francesca.magherini@unifi.it (F.M.); tania.fiaschi@unifi.it (T.F.); matteo.becatti@unifi.it (M.B.); alessandra.modesti@unifi.it (A.M.).
[2]Department of Chemistry, University of Florence, via della Lastruccia 3-13, 50019, Sesto Fiorentino, Firenze, Italy.; lara.massai@unifi.it (L.Ma.).
*Correspondence: tania.gamberi@unifi.it; Tel.: +390552751233 (T.G.); luigi.messori@unifi.it; Tel.: +390554573388 (L.Me.).

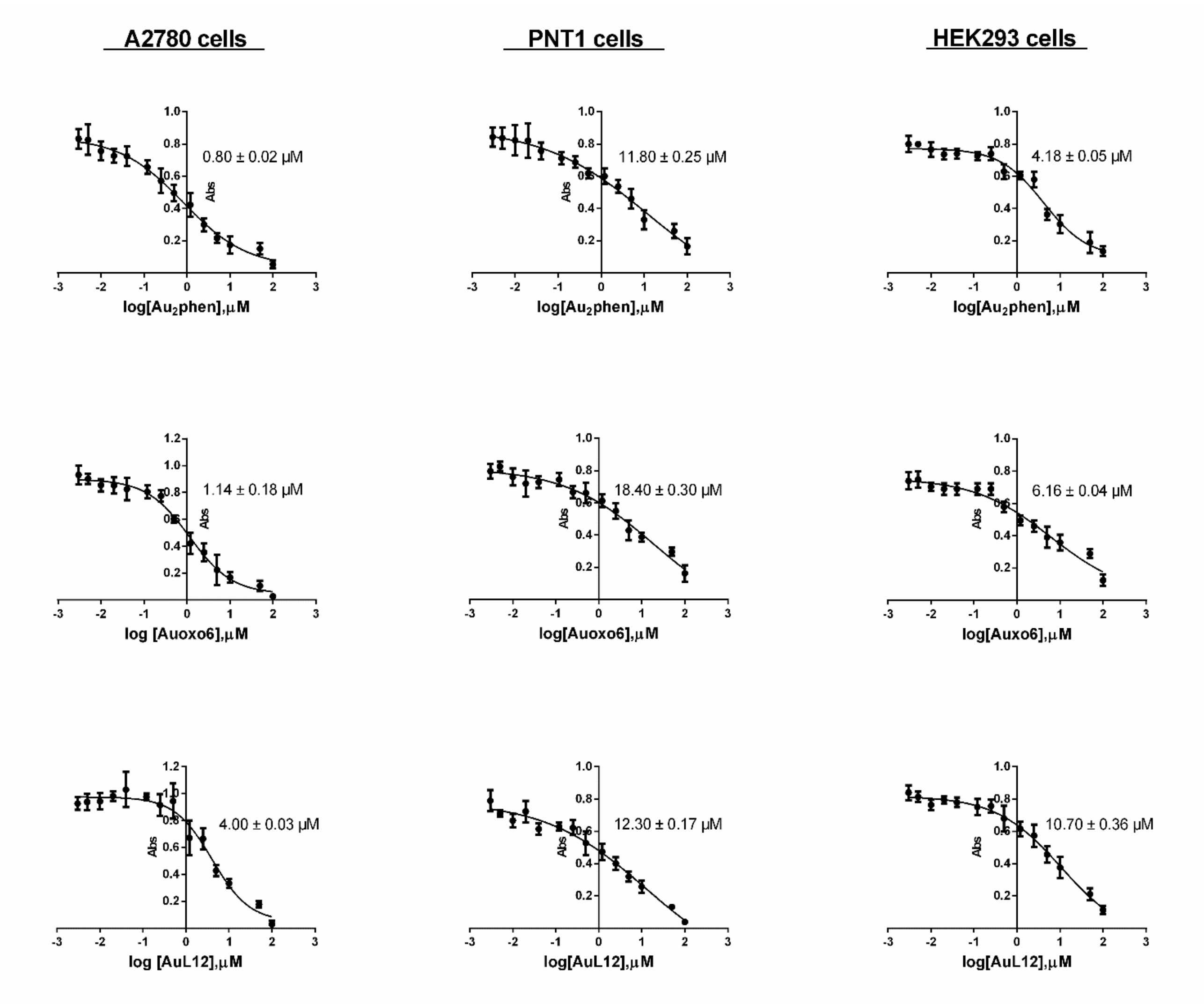


**Figure S1**. Dose-response curves determined using MTT assay. Representative dose –response curves of three independent experiments performed to calculate the half-maximal inhibitory concentration ($IC_{50}$) of the gold compounds Au2phen, Auoxo6 and AuL12 after 72 h on A2780 ovarian cancer cell line and on non-tumor cell lines i.e., HEK (human embryonic kidney) and PNT1 (human normal prostate epithelium) cells. Exponentially growing cells were seeded in 96 well-microplates at a density of $8x10^3$ for 24 h, and then gold compounds were added in fresh RPMI medium at concentrations

ranging from 0.003 to 100 μM, and incubated for 72 h. In the figure are also reported the $IC_{50}$ mean values and the standard deviation of the three independent experiments (see also Table 1 in the manuscript).

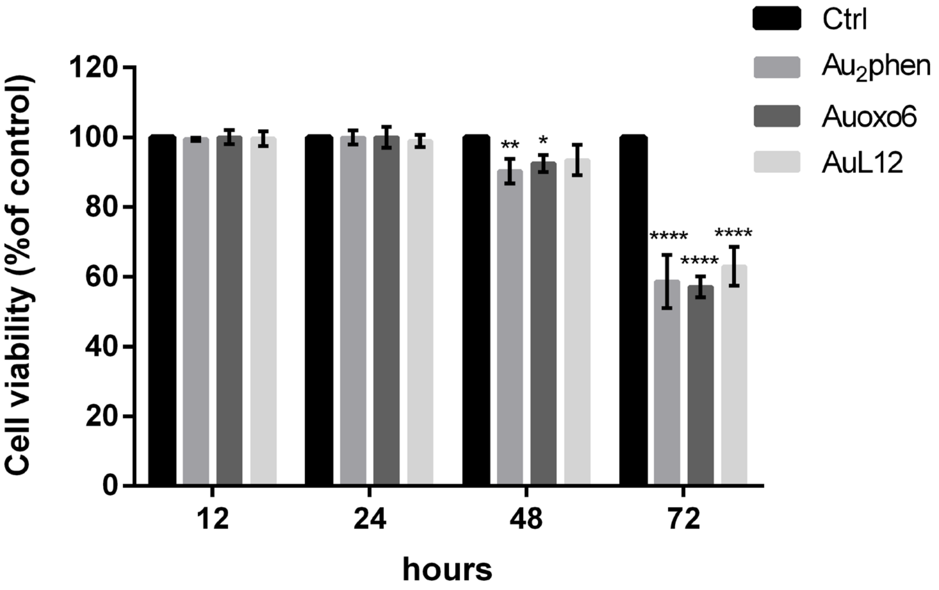


**Figure S2**. Cell viability time course assay upon gold compound treatment. Cell viability of A2780 cancer cells after 12, 24, 48 and 72 h of treatment with the 72 h-$IC_{50}$ doses of each gold compound using MTT assay. The experiment was performed in triplicate. The histogram shows the mean values and standard deviation of the percentage of treated-A2780 viable cells relative to untreated controls. The statistical analysis was carried out using one-way ANOVA test followed by Tuckey's multiple comparisons test using Graphpad Prism v 6.0 (*p<0.05, **p<0.01, ****p<0.0001). (For details, see also Material and Methods, section 2.4. Study of the cytotoxic effects on A2780 cell line).

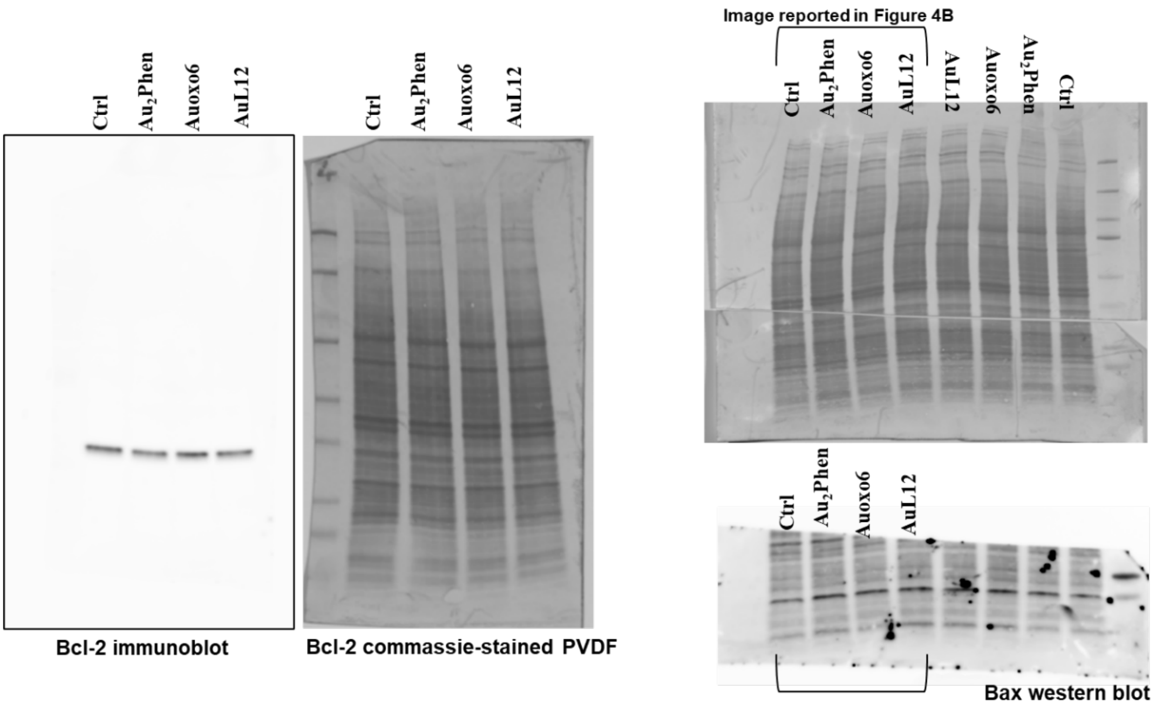


**Figure S3**. Original western blot images of Bcl-2 and Bax content. Original and uncropped western blot images of the representative immunoblots showed in Figure 4B. Their matching coomassie-stained PVDF membranes,used as loading control, are also reported.

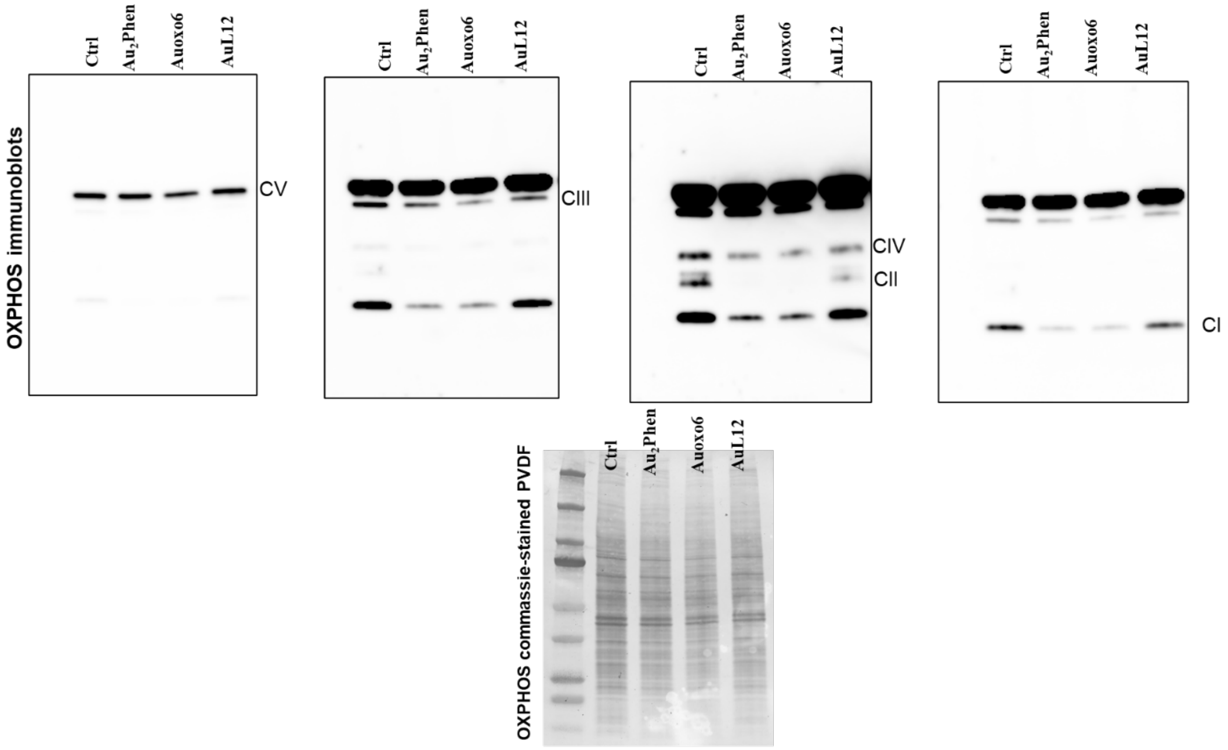


**Figure S4**. Original western blot images of OXPHOS content. Original and uncropped western blot images of the representative immunoblots showed in Figure 5C. Their matching coomassie-stained PVDF membranes,used as loading control, are also reported. Western blot was performed using OXPHOS cocktail (1:1000 dilution, AbCam ab110413) as

reported in Materials and Methods. The immunoreactive bands of each OXPHOS complex were detected at a different exposure time.

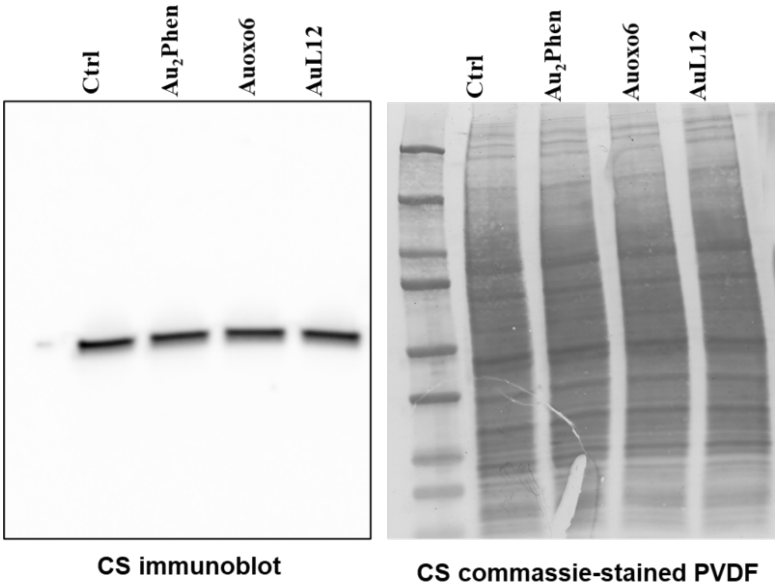


**Figure S5**. Original western blot images of citrate synthase (CS) content. Original and uncropped western blot images of the representative immunoblots showed in Figure 5D. Their matching coomassie-stained PVDF membranes, used as loading control, are also reported.

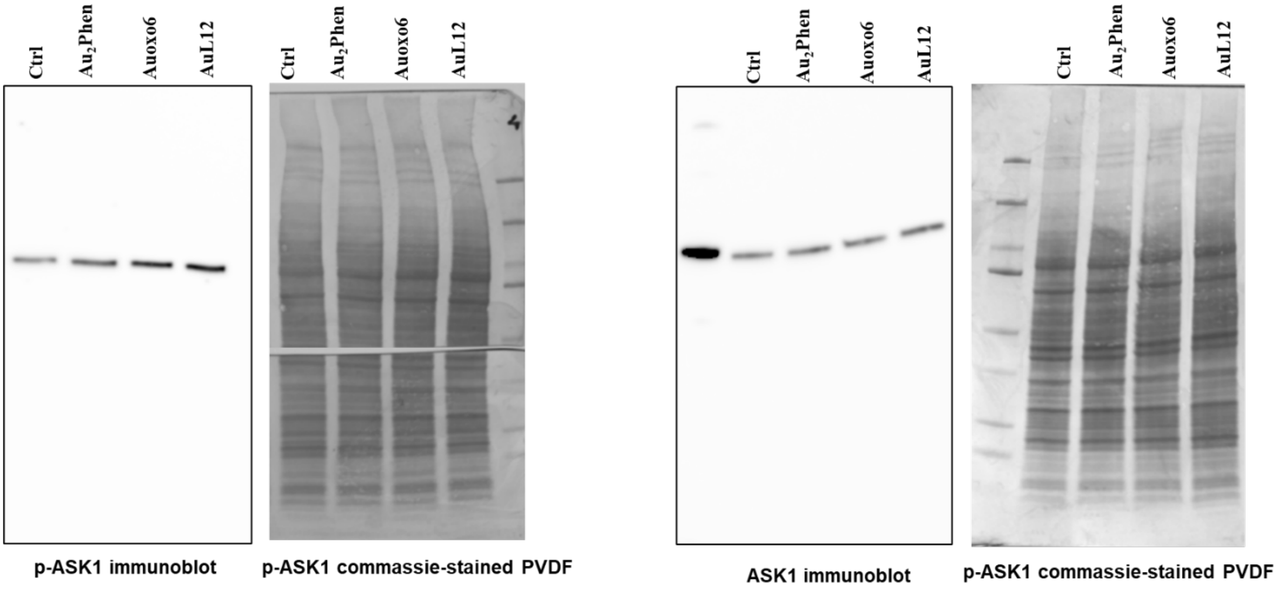


**Figure S6**. Original western blot images of phospho-ASK1 and total ASK1 content. Original and uncropped western blot images of the representative immunoblots showed in Figure 6A. Their matching coomassie-stained PVDF membranes, used as loading control, are also reported.

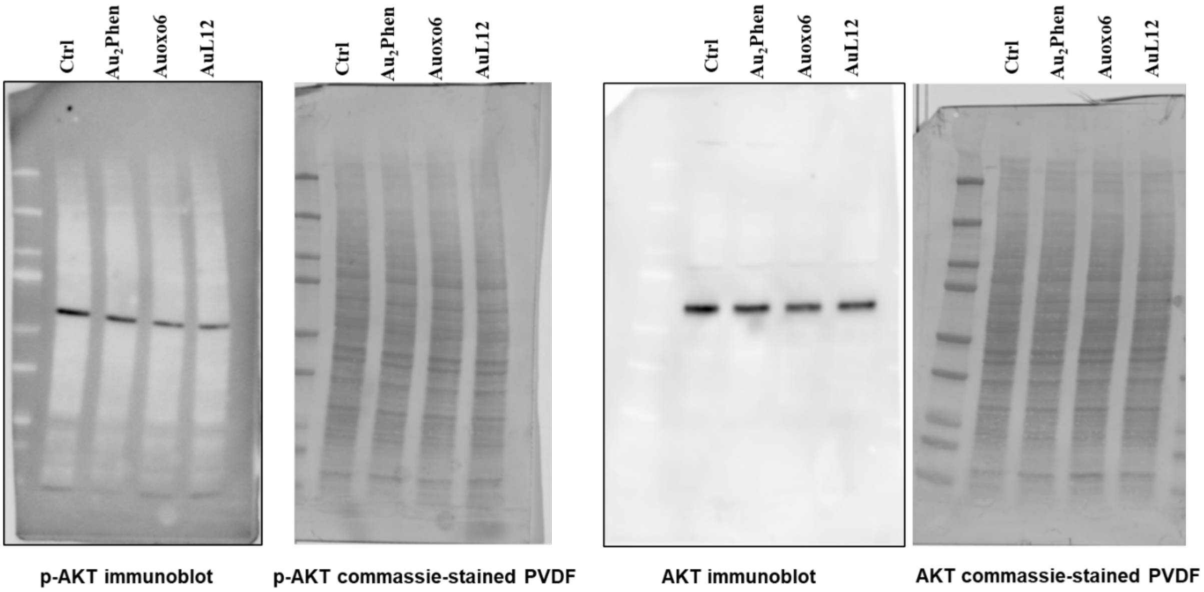


**Figure S7**. Original western blot images of phospho-AKT and total AKT content. Original and uncropped western blot images of the representative immunoblots showed in Figure 6B. Their matching coomassie-stained PVDF membranes, used as loading control, are also reported.
